# Habitat degradation alters trait-based survival in a coral reef fish

**DOI:** 10.1038/s41598-025-22578-y

**Published:** 2025-11-04

**Authors:** Claire M. Moad, Eric P. Fakan, Rohan M. Brooker, Mark I. McCormick

**Affiliations:** 1 ARC Centre of Excellence in Coral Reef Studies, James Cook University, Townsville, QLD 4811 Australia; 2 School of the Environment, University of Queensland, Brisbane, QLD 4072 Australia; 3 Department of Marine Biology and Aquaculture, James Cook University, Townsville, QLD 4811 Australia; 4 Australian Institute of Marine Science, Indian Ocean Marine Research Centre, The University of Western Australia, Crawley, WA Australia; 5 Coastal Marine Field Station, School of Science, University of Waikato, Tauranga, New Zealand

**Keywords:** Reef restoration, Habitat degradation, Coral reef fish, Behaviour, Survival, Climate change, Behavioural ecology, Climate-change ecology, Community ecology, Population dynamics

## Abstract

This study investigates the effect of habitat degradation on the interrelationships between morphological, performance, and behavioural traits and their influence on the survival of juvenile Ambon damselfish (*Pomacentrus amboinensis*). The research combined laboratory measurements with field observations on live or dead-degraded coral reef patches. Fish behavioural traits emerged as key predictors of survival across habitats. Specifically, boldness, bite rate, and maximum distance ventured from the patch reef were key factors. However, the relative importance of specific traits differed between habitats. On live coral, relative lateralization was a significant predictor of survival. In contrast, for fish on dead-degraded coral, routine swimming speed and fast-start response latency were more important. Damselfish in dead-degraded habitats experienced significantly higher mortality. These results indicate that habitat degradation alters the selective pressures on phenotypic traits. This study highlights the importance of considering context-dependent trait selection when predicting how fish communities will respond to changing habitat conditions. Additionally, our findings provide insights for restocking and restoration interventions, suggesting that behavioural conditioning that promotes risk-averse behaviours and predator recognition could improve the survival of reseeded fishes, although further testing within this applied context is required.

**Supplementary Information:**

The online version contains supplementary material available at 10.1038/s41598-025-22578-y.

## Introduction

Understanding how morphological, performance, and behavioural traits influence survival is crucial for predicting how populations will respond to environmental change, and how the relative importance of these traits may shift under changing selective pressures. With habitat quality in global decline^1^, identifying traits that may enhance or decrease survival under degraded conditions could inform predictions of how populations and communities may be reshaped in response. This knowledge also has direct applications for conservation and restoration interventions, particularly with regards to reseeding programs aimed at enhancing populations within degraded systems^2^. Understanding what promotes the survival and success of reseeded organisms is critical for the success of restoration efforts, as ill-informed restoration attempts can be costly^3,4^. Since reseeding usually targets life history bottlenecks, such as the transition from larva to juvenile^5,6^, it is particularly important to understand the drivers of success at these critical periods. Therefore, identifying and/or selecting individuals with traits suited to predicted environmental conditions could improve outcomes for both natural and managed populations.

Theory and research shows that the interrelationships among an organism’s morphological, performance and behavioural traits are key to their survival (e.g^7^^–^^10^). Variation in these traits and interrelationships suggests that organisms are able to adapt to suit their current environment, as long as there is underlying genetic variation influencing these traits. However, understanding the relationship between selection and these potentially interrelated traits is difficult. Arnold^7^ put forward a theoretical framework called the ‘ecomorphological paradigm’ that quantifies how selection acts on suites of characters and their influence on survival and fitness using multivariate selection theory. In this paradigm, morphology is linked to survival and fitness via performance, which is in turn modified and made specific to context by behaviour^7–9,11,12^. This multivariate approach can be used to determine how different characteristics contribute to survival and can be useful regardless of whether the focus is the whole or part of an organism’s lifecycle (e.g^10^^,^^13^).

The phenotypic traits that may offer a selective advantage for surviving can be grouped into three broad categories: morphology, performance and behaviour. These traits can potentially have both independent and interactive effects. For organisms with complex life histories, such as fishes, invertebrates and amphibians, these include morphological traits such as size^14^, locomotory structures^15^, distracting pigmentation (e.g. ocellus/false eye spots^10,16^, growth rate^17^; performance traits such as turning bias (lateralization^18^ or escape kinematics^19^; and behavioural traits (e.g., bold versus shy^20^). Most previous studies have used laboratory experiments to determine the importance of individual traits to survival (e.g^21–24^). This is useful to determine which variables may be of importance, but due to covariance of phenotypic traits it is difficult make conclusions about the role any one factor might play in affecting the survival of an individual^10,25^. Arnold^7^ found that while the effect of morphological variation on performance could be studied in the laboratory, measures of fitness and the effect on survival are best studied in the field, and a combination of laboratory and field studies is required in order to determine the relative importance of interrelated traits that influence fitness and survival^9^. While this combined laboratory and field approach is attractive, it can also be logistically challenging to implement and there are few examples of this approach that exist.

Environmental variability and habitat are key components of the ecomorphological paradigm because many morphological, performance and behavioural traits, along with their interrelationships are context-dependent and vary with environmental conditions^8,26–30^. Organisms must regulate morphological, physiological, performance and behavioural traits in order to cope with local conditions and therefore changes in ambient environmental conditions may alter the expression these traits and as well as their interrelationships^25,31^. Much of the variability in the interrelationships between phenotypic traits is as a result of different environmental conditions^25,32^. Consequently, when habitats degrade, theory suggests that the interrelationships that promote survival may be altered^32^.

Coral reefs are particularly vulnerable to habitat degradation through a variety of local and global scale drivers^33,34^, and as the coral habitat degrades the fish inhabitants are faced with new challenges. Reef degradation may not only affect fish communities through changing resources, but also by altering predator-prey interactions^35–38^. Recent studies have also shown strong links between performance capabilities of fishes and behaviour^10,39,40^, and a number of studies have demonstrated that degraded coral detrimentally affects key aspects of performance that may affect survival (e.g., escape behaviour^39,41^. To date, there have been few attempts for fishes to consider how multiple, interrelated traits are affected by coral degradation, possibly because of the complexities associated with conducting multifactorial, manipulative experiments (e.g^32^^,^^38^^,^^42^^,^^43^).

The present study examined the effect of habitat degradation on the interrelationships among morphological, performance and behavioural traits and their influence on survival of the juvenile Ambon damselfish, *Pomacentrus amboinensis*. Juvenile *P. amboinensis* were reared in two different habitat types (live or degraded coral) and the data was collected in a two-stage experiment: firstly, morphological and performance characteristics were measured in the laboratory for each individual; secondly, these individuals with known characteristics were released onto patch reefs of differing quality (live and degraded coral) that matched their rearing environment, with subsequent behaviour and survival monitored. Boosted classification trees were used to determine the key drivers of survival and whether these differed with habitat quality. These are particularly effective analytical tools as they can fit complex nonlinear relationships to multiple predictor variables and automatically handle interactive effects that are common in ecomorphological relationships^44–46^. Due to the hierarchical links between morphological, performance and behavioural traits with survival^7–10^, the ability to predict survival should be strongest in higher order traits such as behaviour. Therefore, it was expected that the field behavioural measures (boldness, bite rate, space use) will be the best predictors of survival, along with the performance measures of escape response, given their ecological relevance to predator-prey interactions^47^. It was predicted that morphological variables would be less important to survival, due to limited variability in size at this life-stage^48,49^. Understanding how different traits are affected by habitat degradation, and which are selected for under different habitat conditions, is not only crucial for understanding how natural populations and communities will respond to changes in habitat quality, but may also help in developing and refining effective reseeding programs for coral reef fishes. To this end, such information may allow us to select individuals with advantageous traits for the predicted conditions they will experience and maximize the long-term success of restoration efforts in the face of climate change.

## Materials and methods

### Study site and species

The study was conducted at the Lizard Island Research Station and fringing reef on the northern Great Barrier Reef, Australia (14°40’S, 145°28’E). The focal species, the Ambon damselfish, *Pomacentrus amboinensis* (Family: Pomacentridae), is a common component of shallow reef fish communities within the GBR and Indo-Pacific. *P. amboinensis* is one of several coral reef fishes whose risk assessment mechanisms are now known to be compromised by the chemistry emanating from dead-degraded coral^36,37^. Like most aquatic non-mammalian organisms, fish assess and learn about risk through the detection and use of alarm odours that are released when the skin of a conspecific or closely related heterospecifics is damaged^50,51^. Under degraded conditions, the ability of *P. amboinensis* to use alarm odours to assess risk^35^, identify predators [through chemical associative learning or diet cues^52^^,^^53^ or prime their escape behaviour^39^ is compromised. This sensory impairment can disrupt interactions with predators, leading to higher mortality in degraded habitats^54,55^. The mechanism underlying this apparent sensory impairment is unclear, but current evidence suggests that it is most likely due to chemistry emanating from the dead-degraded coral that somehow modifies the activity of the chemical alarm molecules^56,57^.

**Fig. 1:**
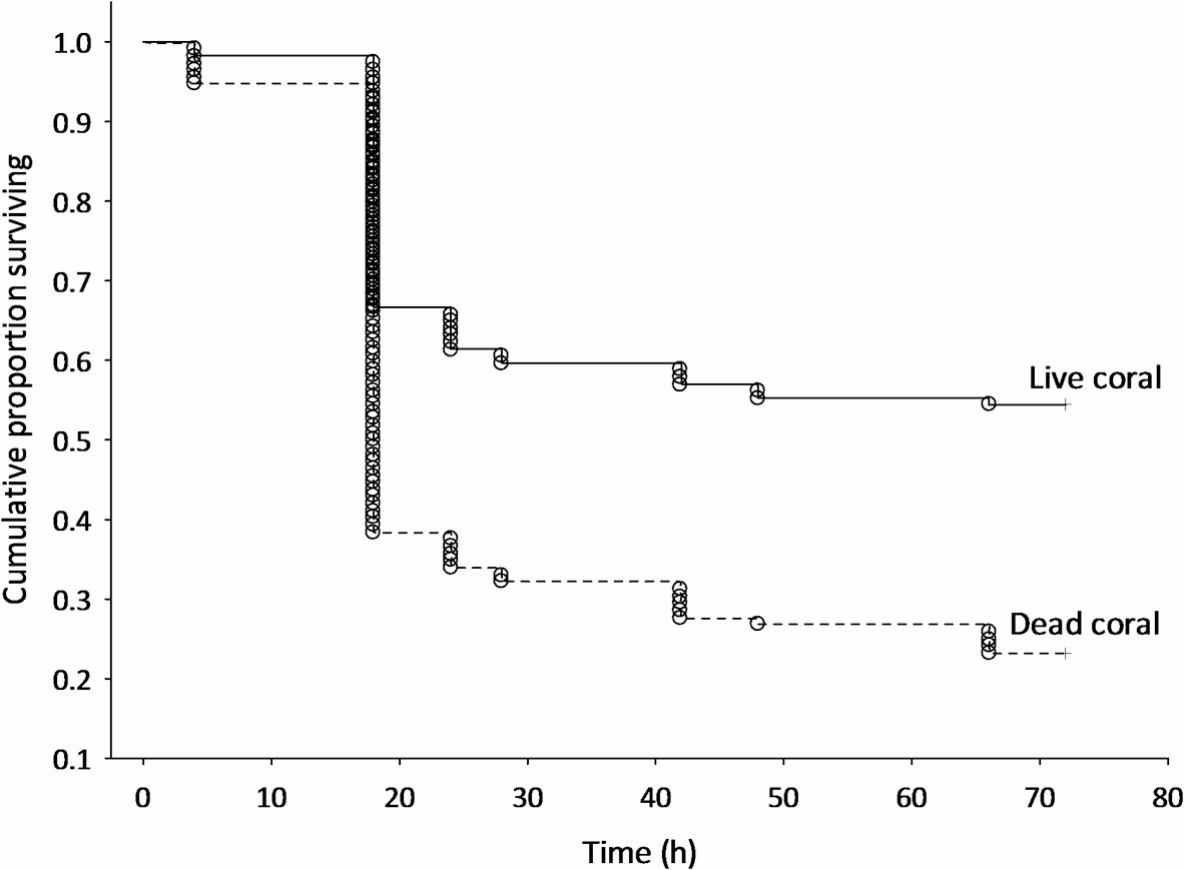
Kaplan-Meier survival curve of the cumulative proportion of surviving *P. amboinensis* over 72 h on patches of live or dead coral (n _live coral_ = 114, n _dead-degraded coral_ = 112). Fish were released at ~ 13:00 h.

Live healthy and dead-degraded *Pocillopora damicornis* colonies were collected from the shallow, fringing reefs around Lizard Island. *Poc. damicornis* is a type of bushy hard coral that is common on the reefs around Lizard Island and the Great Barrier Reef and is a common nursery habitat for fishes^58^. The dead-degraded corals collected had similar topographic/structural complexity to the live corals but were instead covered in a variety of algae, cyanobacteria and sessile invertebrates [see Fig. 1 in McCormick and Lönnstedt^36^. These coral colonies were used both in the treatment tanks in the laboratory and the habitat patches used for field portion of this study and our references to degraded coral habitat in this study refer to this habitat type.

### Experimental overview

Newly metamorphosed, settlement-stage *P. amboinensis*were caught overnight using light traps that were moored in the waters off Lizard Island [small light trap design from Meekan, et al^59^. (see Supplementary Fig. S1 for experiment overview). At this life-stage, the fish are naïve to the reef environmental conditions and reef-associated predators^52^. Once collected from the light traps at dawn, the fish were transported back to the research station in 60 L plastic bins, sorted by species, and held in 30 L aquaria with shelters (PVC pipe pieces) and constant water flow (densities of approximately 30–40 individuals per tank). From these temporary holding tanks a random selection of fish were moved into 30 L treatment tanks housing approximately 20 individuals each. These treatment tanks were established so that they had a continuous supply of aerated seawater that had passed through 30 L header tanks containing either live healthy coral or dead-degraded coral. There were three replicate systems (i.e., a total of six tanks with three per seawater source). Corals and the invertebrates and algae that live on dead-degraded corals thrive best in their natural conditions (i.e., clear, low nutrient, shallow water with high light conditions). To maintain the laboratory treatments so that they best represent what happens in the natural environment we changed the live and dead-degraded coral regularly. The fish were reared in these tanks for three days and fed *Artemia* to satiation twice daily (morning and afternoon). This holding period allowed the fish to recover from the stress associated with the collection process while also being exposed to the treatments throughout. Fish used for the study were randomly selected from the tanks and not all fish were used per tank given the space limitations of the reefs available for stocking (below).

After three days, each individual was put through an array of performance tests and photographed (for morphology, Fig. S2) before being placed on a habitat patch located on the reef edge, where their behaviour was assessed and survival monitored (Fig. S1). The phenotypic variables measured were: (1) body size (standard length and body depth), (2) lateral body area, (3) relative size of the ocellus (false eye spot) – indicator of risk history of the individual, (4) lateralization – the propensity to turn left or right (Fig. S3 for apparatus), (5) routine swimming – measure of activity or exploratory behaviour, (6) fast-start kinematics – startle response needed to evade predator attacks using a repeatable stimulus, (7) behaviour in the field, and (8) survival over three days in the field (see Supplementary files for methods details). Previous research has found that each of these variables may be the focus of selective mortality or that they may be responsive to the magnitude of risk, and thereby are important to survival in reef fishes (Table S1). A total of 226 fish were used in this study (n _live coral_ = 114, n _dead−degraded coral_ = 112). Due to the number of potentially stressful tests being performed on the small juvenile fish in succession, it was important to minimize their stress at each step of the study. Therefore, minimum habituation periods were included before each stage of data collection to reduce any bias caused by the stress of the previous assay. Laboratory assays were always undertaken in the same order, with the least stressful assay (lateralization) being undertaken before more stressful assays (in order: routine swimming, fast-start kinematics, photography; see Fig. S1). Previous physiological studies have shown that juvenile coral reef fishes can recover quickly from stressful situations (an extreme example being exhaustion post-assessment of maximum aerobic capacity^60^).

Once laboratory assays had been completed, fish were individually placed in labelled plastic bags of seawater were stored in 60 L plastic bins (water bath) and covered with a shade cloth to minimize stress. SCUBA divers then released the fish individually onto numbered vacant patch reefs (25 × 20 × 20 cm; one fish per patch) made of either live or dead-degraded coral (in keeping with the habitat they were reared on) and a small cage (30 × 30 × 30 cm cage; 12 mm square mesh) was placed over the patch to allow the fish to habituate to the new environment without the threat of predation. Fish placed on the reefs were randomly chosen under the constraint that the laboratory and field habitats were consistent. Since these fish seldom move between habitat patches immediately after settlement^61^ we did not test for the scenario where they were on the alternative habitat type to that experienced in the laboratory. After 40–60 min, the cages were slowly removed, and fish were assessed for their feeding rates (strikes per minute) and space use over a 3 min period following the protocol of McCormick^61^. Performance trials (lateralization, routine swimming and fast-starts) and photographs were carried out between 08:00 and 12:00 h, and fish were placed onto patches at ~ 13:00 h, with behavioural observations made between 14:00 and 16:00 h.

### Survival

Survival censuses were conducted three times a day (~ 06:30, 13:00 and 16:30) via snorkel for three days post-placement on patches. During the census, each patch was checked for presence or absence of the focal fish. Since fish were not tagged to minimize handling stress, the absence of a fish on the labelled patch reef was assumed to be due to mortality. Our previous studies using tagged fish to quantified emigration on patch reef set-ups similar to that used here have consistently found that movement is very low^61–65^. Because of this research foundation, we believe that equating loss to mortality is a parsimonious assumption within this system. After 72 h (i.e. third day post-placement on patches), if the individual was still alive and present on the patch, it was recorded as being present before being removed from the patch.

### Statistical analyses

All statistical analyses were completed using TIBCO^®^ Statistica™ (Version 13.3.0).

#### Data screening and preliminary analysis

Descriptive statistics (mean, standard deviation, minimum and maximum, variance; Supplementary Table S1) were computed for each variable. While regression tree analyses do not require normality, analyses often perform better when variables are normalised^45^, so a number of the variables were transformed prior to analysis (see Table S1 for transformation details).

The lateralization variables and other field behavioural variables (boldness, bite rate, maximum distance ventured) were slightly non-normal, however did not respond well to transformations and hence the raw data was used in analyses. Correlations between variables were used to check for co-linearity (Table S2). There was a strong correlation between both response speed and response latency with the distance the fish was from the stimulus and therefore the residuals against distance to stimulus were computed for these variables to remove the effect of variable distance. Distance to the stimulus was then dropped from analyses. Where independent variables were similar measures of the same phenotype (e.g. speed and distance moved in both routine swimming and fast-start variables) only speed was included as it was more ecologically relevant (i.e., how fast an individual swims away is more ecologically relevant than how far it swims). Of the two lateralization variables measured, only relative lateralization was used in subsequent analyses as it was more normally distributed. A principal component analysis was undertaken on the three field behavioural variables to produce a unified variable that represented the propensity to take risk (i.e., boldness)^66^. All three variables (bite rate, total distance moved and maximum distance ventured) had high and similar loadings on the first axis (0.78, 0.88, 0.8 respectively), which represented 68% of the variation in behaviour among fish (see Fig. S4 for a biplot). This left 11 variables that were used in all further analyses: (1) standard length, (2) body depth, (3) ocellus/eye area, (4) lateral body area, (5) routine swimming speed, (6) routine swimming maximum speed, (7) response speed, (8) response maximum speed, (9) response latency, (10) relative lateralization, (11) boldness (principle component 1 on the field behavioural variables).

#### Survival analysis

The field survival data was converted into time-to-loss data, where there were two possible events: complete (i.e., individual died) and censored (i.e., individual was still present at 72 h post-placement on patch and removed). Kaplan-Meier analysis was used to construct cumulative survival plots to compare survival trajectories between the live coral and dead-degraded coral habitat and Cox-Mantel (log rank) test was used to determine whether these curves are significantly different.

#### Boosted classification trees

Boosted classification trees were used to relate the selected 11 variables to whether the fish survived on the habitat patches for each treatment. Tree analyses can explain variation of a single response variable, either categorical (classification trees) or numeric (regression trees), by repeatedly splitting the data into more homogenous groups using combinations of explanatory variables^67^. They are ideal for analysis of complex ecological data since they are generally fairly flexible and robust and can handle complex nonlinear relationships with potential high-order interactions and missing values^44,45,67^. Boosting is a method for improving model accuracy on the basis that it is easier to find and average many simple models than a single highly accurate model^44,45,68^. Therefore boosted classification trees will average the relative importance of each predictor variable to response by averaging it over multiple trees, significantly improving the accuracy of predictions^44,45^. The relative predictor importance of each variable to survival was computed (using the sums of squares regression), giving a value from 0 to 1, with 1 being the most important, and then compared between the two treatments. Because of the rapid loss of fish from the patch reefs, survivorship was based on whether they were present at the census 24 h post-placement on the patch reefs. Replicates for each habitat at this time and used in the analysis were (alive/dead fish): live coral 70/44; dead-degraded coral 38/74. Analysis was completed using the Data Miner package from Statistica™ (Version 13.5.0.17).

#### Univariate analysis

Based on the variables with higher predictive importance to survival, or large differences in importance between the two treatments, univariate analyses were conducted on key variables to further explore the nature of the differences found by multivariate analyses. Two-factor analyses of variance (ANOVA, type III sums of squares) were used to compare means of key predictor variables with fixed factors: Habitat (live coral, dead-degraded coral) and Survival (survived or died at 24 h). Assumptions of ANOVA were examined using residual analyses prior to analysis. Results of these analyses are interpreted cautiously due to the non-independent nature of the key variables (i.e., undertaking multiple dependent tests thereby elevating Type I error rates). Effect sizes are given as partial eta squared values (η_p_^2^).

## Results

### Survival

Survival trajectories of *P. amboinensis* individuals were significantly different between those on live coral patches compared with those placed on dead-degraded coral patches (Cox-Mantel test: C = 4.93, *p* < 0.0001). Survival was significantly lower on the dead-degraded coral patches with only 23% of individuals surviving until the 72 h census, in comparison to those on healthy live coral patches where approximately double the percentage of *P. amboinensis* survived until the 72 h census (54%; Fig. 1). For both habitats, a significant proportion of individuals were absent (i.e. died) at the 18 h census, with approximately 30% of individuals dying overnight from the live coral, and twice as many dying on the dead-degraded coral patches (Fig. 1).

### Boosted classification analysis

**Fig. 2:**
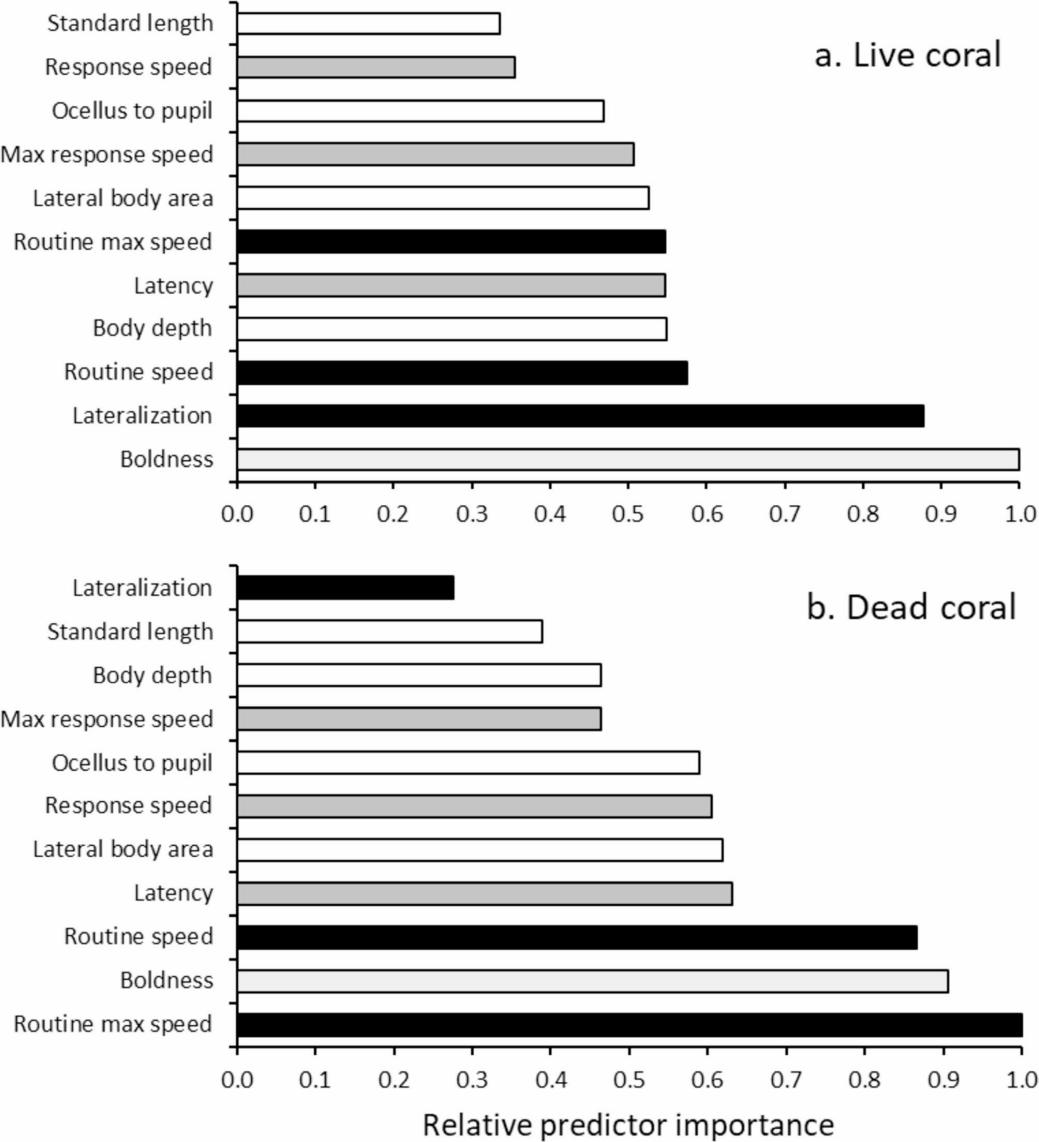
Relative importance of interrelated morphological, performance and behavioural traits to survival of *P. amboinensis* within the first 24 h for (**a**) live coral and (**b**) dead-degraded coral habitats. Bar colours: white – morphology; light grey – behaviour; grey – fast-start response; black – routine swimming and lateralization.

The relative importance of the various phenotypic variables to predicting survival of *P. amboinensis* differed between the live and dead-degraded coral treatments (Fig. 2). For individuals in the live coral treatment, the composite boldness variable and lateralisation score were the best predictors of survival (Fig. 2a). The least important were standard length and response speed to the startle stimulus. The morphological variables, routine and the fast-start kinematic variables were less important to predicting survival in the live coral treatment (Fig. 2a). In contrast, for those in dead-degraded coral, routine swimming variables (average speed and max speed) and their boldness score were most important to survival. Interestingly, the degree of lateralization, which was so important for fish on live coral, was the least important variable for fish on dead-degraded coral. The morphological variables (standard length, body depth, ocellus to pupil and lateral body area) were all less important for fish that survived on dead-degraded coral (Fig. 2b).

### Univariate analysis

**Fig. 3:**
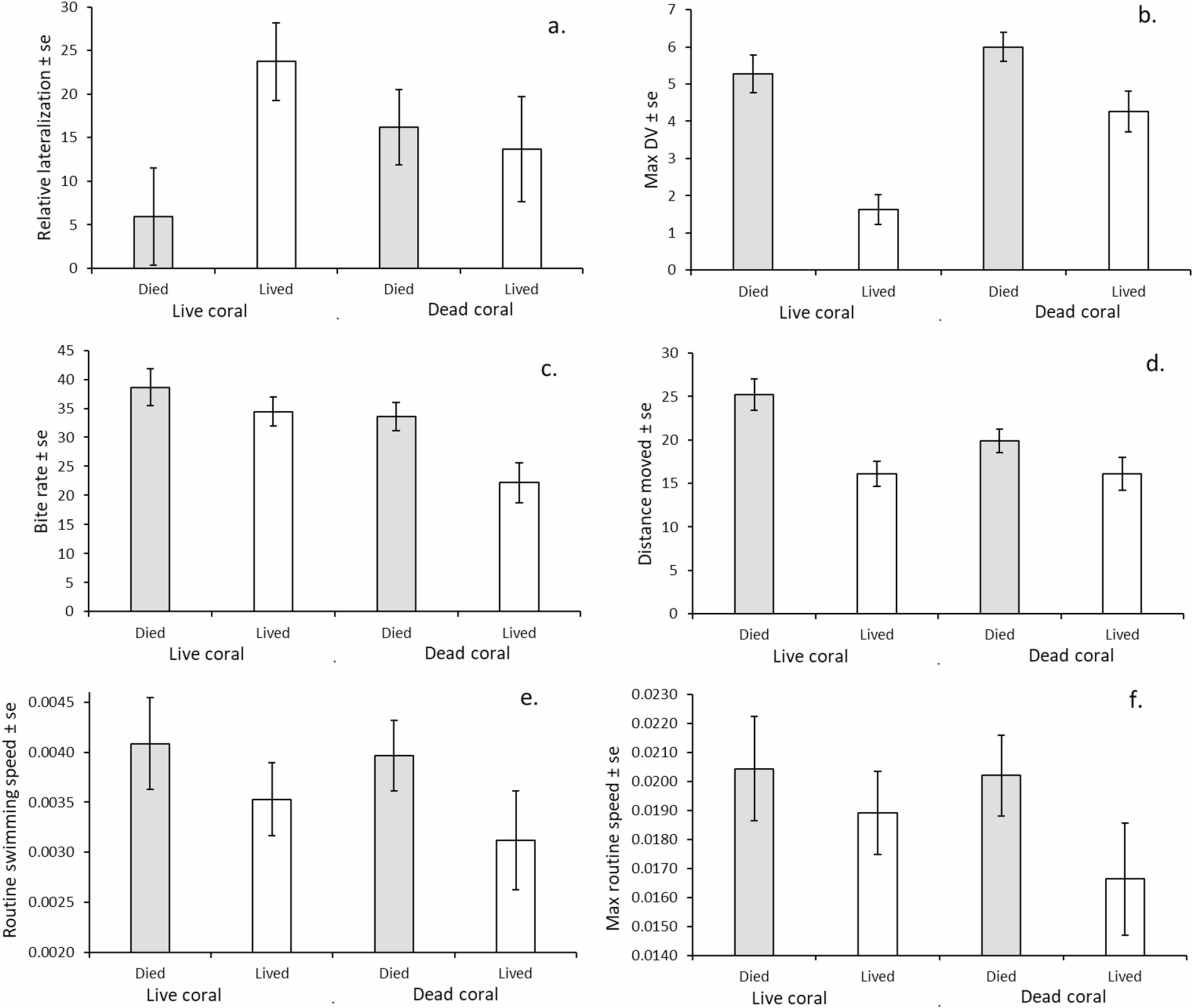
Univariate analysis of key phenotypic variables of juvenile Ambon damselfish (*Pomacentrus amboinensis*) compared between habitat type (live or dead-degraded coral) and survival at 24 h post-placement on patch reefs (died = grey; survived = white). The variables compared: (**a**) relative lateralisation score, (**b**) maximum distance ventured (Max DV, cm), (**c**) bite rate (bites/3min), (**d**) total distance moved (cm), (**e**) routine swimming speed (m/s) and (**f**) maximum routine swimming speed (m/s). Mean ± standard error displayed (n _live coral, died_ = 44, n _live coral, survived_ = 70, n _dead-degraded coral, died_ = 74, n _dead-degraded coral, survived_ = 38).

Relative lateralization was one of the key predictors of survival for fishes on live coral patches, and univariate analysis showed that there was a significant interactive effect between habitat and survival (ANOVA: F_1, 222_ = 3.93, *p* = 0.048), although the effect size is low (η_p_^2^ = 0.017). Fish that survived for 24 h on live coral were significantly more lateralized than those that died from the same treatment and individuals from the dead-degraded treatment (Fig. 3a). The mean relative lateralization of individuals in the degraded coral treatment was similar regardless of whether they lived or died (Fig. 3a).

Due to the importance of the composite boldness score to survival in both live and dead-degraded coral, the variables that made up the PCA score were examined in more detail. Of these three variables, only maximum distance ventured (Fig. 3b) had a significant interaction between factors (F_1,222_ = 4.27, *p* = 0.04, η_p_^2^ = 0.019). Fish that survived on live coral remained closer to their patch reef with a mean maximum distance ventured of 1.62 cm, compared to those that died from the same habitat, which ventured more than 3.5 times further from their patch (5.27 cm). All fish on dead-degraded coral patches ventured further from shelter than those of live coral patches, regardless of whether they survived the first 24 h (survived, 4.26 cm; died, 6.00 cm) (Fig. 3b).

The other field behavioural variables (bite rate and total distance moved) were significantly affected by treatment and/or survival. Lower mean bite rates were observed for fishes that ended up surviving in both habitats (F_1, 222_ = 6.13, *p* = 0.014, η_p_^2^ = 0.027) and there was also a significant habitat effect (F_1, 222_ = 9.998, *p* = 0.002, η_p_^2^ = 0.043). Surviving fish on the dead-degraded coral patches had a lower mean bite rate than surviving fish on the live coral patches, while there were similar mean bite rates observed for fish that died irrespective of habitat (Fig. 3c). The total distance moved within 3 min differed depending on whether fish lived or died (F_1,222_ = 15.68, *p* < 0.0001, η_p_^2^ = 0.07; Fig. 3d), and there was a trend for a difference in activity between habitats (F_1,222_ = 3.37, *p* = 0.07, η_p_^2^ = 0.015). The other variables of average and maximum routine swimming speed mirrored the trends shown in the other behavioural variables, but with more variance (Fig. 3e, f).

## Discussion

This is the first study to explore whether the variables that influence survival of coral reef fishes at the crucial early life history stage following settlement differ when their coral habitat dies and degrades. As predicted, there was a significant decrease in survival of *P. amboinensis* on the degraded coral patch reefs in comparison to live coral. Interestingly, there were differences in the variables that related to fish survival within the first 24 h between live and dead-degraded coral, but there was also consistency in the importance of variables associated with the propensity to take risk. In keeping with Arnold’s^7^ ecomorphological paradigm^8,9^ it was the behavioural traits assessed in the field that were consistently important in determining survival, regardless of habitat.

The results confirmed that *P. amboinensis* survival was affected by habitat degradation, with individuals in the dead-degraded coral treatment experiencing 77% mortality in their first 72 h on the reef, in comparison to 54% mortality for those on live coral. Similar results have been obtained for *P. amboinensis*in earlier studies. Lönnstedt, et al^56^. observed 75% increase in mortality for individuals on dead-degraded coral patches in comparison to those released onto live coral patches. Similarly, McCormick^61^ found survival rates were three times higher for *P. amboinensis* on live coral than those on degraded or thermally bleached coral. All juvenile reef fish experience high levels of early post-settlement mortality due to predation when they first settle on the reef, forming a population bottleneck^69,70^. Mortality rate then declines as the fish gain experience on the reef and learn risk^52,71,72^, therefore the initial mortality experienced by a cohort plays a key role in shaping populations.

Behavioural traits involving activity and the willingness to take risk were important in predicting survival chances in both habitats. Regardless of whether fish were on live or dead-degraded coral, individuals that were more active and travelled furthest from shelter had a higher probability of dying within the first day on the reef. Theory suggests that conservative and risk-adverse behaviours that minimize the risk of predation may be favoured by selection at this early life stage^61,72^ and consequently these traits are useful predictors of survival. How far an individual ventured from the patch (maximum distance ventured) was particularly important in predicting survival of fish on live, healthy coral patches, where surviving individuals remained significantly closer to their patch than those that died or those on dead-degraded coral patches. McCormick^61^ also found that *P. amboinensis* on live coral patches remained closer to their patch, while individuals on bleached or dead-degraded coral patches tended to sit higher in the water column and venture further from their patch, increasing their susceptibility to predation. Our finding of the key importance of behavioural attributes supports the ecomorphological paradigm that predicts that behavioural traits have the strongest effect on survival and fitness at this life stage, by acting as a filter between lower order variables (morphology, performance) and survival^7–9^.

Relative lateralization was identified as an important variable in predicting fish survival on live coral, but was one of the least important variables for fish on dead-degraded coral patches. Surviving individuals on live coral patches were more lateralized than any other group, with mean relative lateralization scores four times that of their counterparts that died from the same treatment. Being highly lateralized can be advantageous to survival as it may increase neural capacity allowing individuals to perform more than one task at a time^73,74^. In the case of escape behaviour, being highly lateralized in one direction or another means that an individual may be quicker to react to a predator approaching on that side, or be able to escape from predators more quickly by not having to think about which direction to turn^18,75,76^. Individuals that survived on the live coral patches had a higher mean lateralization score and the high relative importance of this trait in this habitat type suggests it was advantageous in their ability to escape from predators. Lateralization is also closely associated with background risk, with fish in high-risk environments (e.g. increased predators) more highly lateralized than individuals in low risk environments, suggesting that in high-risk environments higher behavioural lateralization may provide a selective advantage to survival^75,77,78^. Since habitat degradation is known to detrimentally affect risk assessment in many species through its effect on the utility of alarm cues (e.g^35^^,^^37^), it is possible that the dead-degraded coral environment is interfering with an individual’s ability to detect high risk conditions and in so doing is negating selection on this trait.

Routine swimming speed was an important predictor of survival for *P. amboinensis* in both the live and dead-degraded coral treatments, however univariate analysis showed no differences in mean routine swimming speed with treatment or survivorship. Routine swimming, as a measure of general activity, has been previously associated with survival in reef fishes as it is related to their foraging and exploratory behaviour^79,80^. It can also alter an individual’s risk of encountering predators; if an individual moves around a lot it may be more conspicuous to predators^54,79^. Interestingly, an experimental study that allowed fish to associate with dead-degraded or live coral for 6 weeks found that routine swimming can be affected by degraded habitats, with *P. amboinensis* individuals reared in the seawater that had passed over dead-degraded coral moving 13% more than those reared in healthy, live coral^38^. The authors argued that this increased activity may contribute to increased mortality for fishes on degraded reefs^81^. The difference in time-scales between these studies (3 days versus 6 weeks) may explain why no significant effect of habitat degradation on routine swimming was observed in the current study, as longer periods of exposure to the degraded coral may promote a greater effect on this trait. The absence of any statistical difference between the two habitat types, suggests that while routine swimming is a useful predictor of survival, it is likely to be working in combination with variables like boldness in a more general risk exposure framework.

Fast-start response latency was a moderately important trait for predicting survival of *P. amboinensis* in both live and dead-degraded habitats. Latency is often associated with survival as it plays a key role in determining the outcome of predator-prey interactions, with respect to feeding success of the predator or escape and survival of the prey^82^. A recent field study found response latency to be the best predictor of survival for another juvenile damselfish, *P. chrysurus*^10^. In the current study, there was no difference in latency between habitat types when compared using univariate analysis. This was in keeping with laboratory studies that tested the effects of habitat degradation on the fast-start performance under risk^83^. These studies found that latency was not affected by habitat degradation for three species of juvenile damselfish with different associations with live coral^39^, or by the level of structural complexity of the environment^84^. The finding of a consistent importance of response latency in both healthy and degraded habitats emphasises the importance of the behavioural component to the fast start response, as latency has been couched as the behavioural trade-off of risk versus energetic cost^85^.

Despite being some of the most studied covariates in previous survival studies, the current study found that morphological variables were some of the least important predictors of survival. Many previous studies have shown that post-settlement mortality in reef fish is size selective and that predators will preferentially feed upon smaller (e.g^62^^,^^87^^–^^89^) or larger prey^87,90^. However, in the current study there was a low coefficient of variance for body size (~ 5%) suggesting low phenotypic variance in these traits. This is commonplace for many fish species within a particular settlement pulse^48,49^. As a result of lower variance, there is reduced chance for selection to act upon particular traits, resulting in the low relative importance of morphological variables in general to survival at this life stage. While the size range incorporated in the present study was ecologically relevant to natural settlement, there is a general finding of a fundamental and cumulative importance of size advantages^91,92^. For reef fishes at this transitional life stage a less than 10% difference in length can drastically improve the probability of surviving the early intense mortality pressure^93^. This suggests that future studies that focus on optimizing the characteristics of reseeded propagules should test the efficacy of other traits against an enhanced size range, as size is always easy to bolster by simply holding offspring for a short period.

This was one of the first studies to look at the effect of interrelationships between morphological, performance and behavioural traits in a coral reef fish on survival in the field in the context of habitat degradation. In both habitat types (live and dead-degraded coral), survival of *P. amboinensis* was best predicted by behavioural characteristics and that selection favoured a shy, more conservative phenotype (i.e. reduced bite rate and boldness, which did not swim as far from patches). There are two mechanisms by which behaviour influences survival: firstly, it determines how an organism uses space and that may influence its vulnerability to predators (e.g^23^^,^^87^); and second, it modifies the relationships between morphological and performance variables by making them specific to context per the ecomorphological paradigm^7–9^. However, there were also major differences in the relative importance of specific variables to survival of *P. amboinensis* in live coral versus degraded coral patches. This suggests that different selective pressures that favour different traits are operating in healthy coral habitats as opposed to degraded habitats. Interestingly, many of the traits that were affected by habitat degradation in this study, have also been associated with how individuals perceive risk, which is known to be affected for *P. amboinensis* in degraded habitats (e.g^35^^,^^55^^,^^61^^,^^83^), and therefore this may be a driving mechanism behind which traits are important to survival. Understanding which traits are selected for under different habitat conditions will enable better predictions of how habitat degradation may affect and shape future reef fish communities.

In addition, given the increasing interest in the potential of reseeding coral reef fishes as a stock management and restoration intervention, understanding the behavioural, morphological, and performance traits that mediate survival under different habitat conditions and contexts may assist in the development and refinement these methods^94^. Based on the findings of this study, we recommend that restoration programs incorporating reseeding prioritise behavioural training to promote risk-averse behaviours before release. To date, behavioural conditioning has not been trialled in reseeding programs on coral reefs. Recent research has shown that chemical cues from degraded coral can hinder the development of neophobia in settling damselfish^55^ while odours from dead-degraded coral and certain algae can impair alarm cue recognition, a critical component of predator learning^37,57^. Training fish to recognize local predators has been shown to significantly improve survival in damselfish^52^ and salmonids^95^, and this learning can extend to multiple predator species through generalisation^96,97^. The initial survival advantage conferred by predator training may allow fish to later rely on direct experience^98^, publicly available information^99^, or dietary cues^53^ to refine their risk assessments. Additionally, as holding fish prior to release naturally increases body size, it may provide an added survival advantage through behavioural covariates linked to size^38^. Future research may therefore benefit from assessing the relative efficacy of behavioural training versus size amplification in reseeding efforts to optimise survival outcomes.

## Supplementary Information

Below is the link to the electronic supplementary material.

Supplementary Material 1

## Data Availability

Data are available at Figshare; doi: 10.6084/m9.figshare.29176481.

## References

[1] Graham, N. A. J., Benkwitt, C. E. & Jones, H. P. Species eradication for ecosystem restoration. *Curr. Biol.* **34**, R407–R412. 10.1016/j.cub.2024.02.033 (2024). 38714173 10.1016/j.cub.2024.02.033

[2] Shaver, E. C. et al. A roadmap to integrating resilience into the practice of coral reef restoration. *Global Change Biol.* **28**, 4751–4764 (2022). 10.1111/gcb.16212 PMC9545251 35451154

[3] Crowe, T. P. et al. Experimental evaluation of the use of hatchery-reared juveniles to enhance stocks of the Topshell *Trochus niloticus* in Australia, Indonesia and Vanuatu. *Aquaculture* **206**, 175–197 (2002).

[4] Hamasaki, K., Takeuchi, H., Shiozawa, S. & Teruya, K. Effects of acclimation to the natural environment before release on retention rate, feeding condition and predation of hatchery-reared juveniles of the coral trout *Plectropomus leopardus* released on a coral reef. *Bull. Jap. Soc. Sci. Fish.* **70**, 2–30 (2004).

[5] Harrison, P. L., dela Cruz, D. W., Cameron, K. A. & Cabaitan, P. C. Increased coral larval supply enhances recruitment for coral and fish habitat restoration. *Front. Mar. Sci.* **8**, 750210 (2021).

[6] Heenan, A., Simpson, S. D., Meekan, M. G., Healy, S. D. & Braithwaite, V. A. Restoring depleted coral-reef fish populations through recruitment enhancement: a proof of concept. *J. Fish. Biol.* **75**, 1857–1867 (2009). 20738653 10.1111/j.1095-8649.2009.02401.x

[7] Arnold, S. J. Morphology, performance and fitness. *Am. Zool.* **23**, 347–361 (1983).

[8] Garland, T. Jr & Losos, J. B. *Ecological Morphology: Integrative Organismal Biology* (eds Wainwright, P. C. & Reilly, S. M.) 240–302 (University of Chicago Press, 1994).

[9] Orr, T. & Garland, T. Complex reproductive traits and whole-organism performance. *Integr. Comp. Biol.* **57**, 407–422 (2017). 28859419 10.1093/icb/icx052

[10] McCormick, M. I., Fakan, E. & Allan, B. J. M. Behavioural measures determine survivorship within the hierarchy of whole-organism phenotypic traits. *Funct. Ecol.* **32**, 958–969 (2018).

[11] Storz, J. F., Bridgham, J. T., Kelly, S. A. & Garland, T. Jr Genetic approaches in comparative and evolutionary physiology. *Am. J. Physiology-Regulatory Integr. Comp. Physiol.* **309**, R197–R214 (2015). 10.1152/ajpregu.00100.2015 PMC4525326 26041111

[12] Lailvaux, S. P. & Husak, J. F. The life history of whole-organism performance. *Q. Rev. Biol.* **89**, 285–318 (2014). 25510077 10.1086/678567

[13] Lachambre, S. et al. Relationships between growth, survival, physiology and behaviour—A multi-criteria approach to *Haliotis tuberculata* phenotypic traits. *Aquaculture* **467**, 190–197 (2017).

[14] Perez, K. O. & Munch, S. B. Extreme selection on size in the early lives of fish. *Evolution: Int. J. Org. Evol.* **64**, 2450–2457 (2010). 10.1111/j.1558-5646.2010.00994.x 20298462

[15] Langerhans, R. B. Morphology, performance, fitness: functional insight into a post-Pleistocene radiation of mosquitofish. *Biol. Lett.* **5**, 488–491 (2009). 19411270 10.1098/rsbl.2009.0179 PMC2781922

[16] Lönnstedt, O. M., McCormick, M. I. & Chivers, D. P. Predator-induced changes in the growth of eyes and false eyespots. *Sci. Rep.* **3**, 2259 (2013). 23887772 10.1038/srep02259 PMC3722912

[17] Hoey, A. S. & McCormick, M. I. Selective predation for low body condition at the larval-juvenile transition of a coral reef fish. *Oecologia* **139**, 23–29 (2004). 14767752 10.1007/s00442-004-1489-3

[18] Bisazza, A., Facchin, L., Pignatti, R. & Vallortigara, G. Lateralization of detour behaviour in poeciliid fish: the effect of species, gender and sexual motivation. *Behav. Brain. Res.* **91**, 157–164 (1998). 9578448 10.1016/s0166-4328(97)00114-9

[19] Domenici, P. & Hale, M. E. Escape responses of fish: a review of the diversity in motor control, kinematics and behaviour. *J. Exp. Biol.* **222**, jeb166009 (2019). 31534015 10.1242/jeb.166009

[20] Dingemanse, N. J., Kazem, A. J., Reale, D. & Wright, J. Behavioural reaction norms: animal personality meets individual plasticity. *Trends Ecol. Evol.* **25**, 81–89 (2010). 19748700 10.1016/j.tree.2009.07.013

[21] Irschick, D. J., Meyers, J. J., Husak, J. F. & Le Galliard, J. F. How does selection operate on whole-organism functional performance capacities? A review and synthesis. *Evol. Ecol. Res.* **10**, 177–196 (2008).

[22] Garland, T. *Quantitative Genetic Studies of Behavioral Evolution* (eds Boake, C. R. A.) 251–277 (University of Chicago Press, 1994).

[23] Holmes, T. H. & McCormick, M. I. Influence of prey body characteristics and performance on predator selection. *Oecologia* **159**, 401–413 (2009). 19018572 10.1007/s00442-008-1220-x

[24] Mihalitsis, M. & Bellwood, D. R. A morphological and functional basis for maximum prey size in piscivorous fishes. *PloS One*. **12**, e0184679 (2017). 28886161 10.1371/journal.pone.0184679 PMC5590994

[25] Garland, T., Downs, C. J. & Ives, A. R. Trade-offs (and constraints) in organismal biology. *Physiol. Biochem. Zool.* **95**, 82–112 (2022). 34905443 10.1086/717897

[26] Rausher, M. D. The measurement of selection on quantitative traits: biases due to environmental covariances between traits and fitness. *Evolution* **46**, 616–626 (1992). 28568666 10.1111/j.1558-5646.1992.tb02070.x

[27] Stinchcombe, J. R. et al. Testing for environmentally induced bias in phenotypic estimates of natural selection: theory and practice. *Am. Nat.* **160**, 511–523 (2002). 18707526 10.1086/342069

[28] Dunson, W. A. & Travis, J. The role of abiotic factors in community organization. *Am. Nat.* **138**, 1067–1091 (1991).

[29] Huey, R. B. Physiological consequences of habitat selection. *Am. Nat.* **137**, S91–S115 (1991).

[30] Killen, S. S., Adriaenssens, B., Marras, S., Claireaux, G. & Cooke, S. J. Context dependency of trait repeatability and its relevance for management and conservation of fish populations. *Conserv. Physiol.* **4**, cow007 (2016). 27382470 10.1093/conphys/cow007 PMC4922260

[31] Wingfield, J. C. *Endocrinology of Social Relationships* (eds P Ellison & P Gray) 74–94 (Harvard University Press, 2009).

[32] Killen, S. S., Marras, S., Metcalfe, N. B., McKenzie, D. J. & Domenici, P. Environmental stressors alter relationships between physiology and behaviour. *Trends Ecol. Evol.* **28**, 651–658 (2013). 23756106 10.1016/j.tree.2013.05.005

[33] Chaudhary, C., Richardson, A. J., Schoeman, D. S. & Costello, M. J. Global warming is causing a more pronounced dip in marine species richness around the equator. *PNAS* **118**, e2015094118 (2021). 33876750 10.1073/pnas.2015094118 PMC8054016

[34] De’ath, G., Fabricius, K. E., Sweatman, H. & Puotinen, M. The 27-year decline of coral cover on the great barrier reef and its causes. *PNAS* **109**, 17995–17999 (2012). 23027961 10.1073/pnas.1208909109 PMC3497744

[35] Lönnstedt, O. M., McCormick, M. I. & Chivers, D. P. Degraded environments alter prey risk assessment. *Ecol. Evol.* **3**, 38–47 (2013). 10.1002/ece3.388 PMC3568841 23403754

[36] McCormick, M. I. & Lönnstedt, O. M. Disrupted learning: habitat degradation impairs crucial antipredator responses in naive prey. *Proc. R. Soc. B* **283**, 20160441 (2016). 27170715 10.1098/rspb.2016.0441 PMC4874716

[37] Ferrari, M. C. O., McCormick, M. I., Allan, B. J. M. & Chivers, D. P. Not equal in the face of habitat change: closely related fishes differ in their ability to use predation-related information in degraded coral. *Proc. R. Soc. B* **284**, 20162758 (2017). 10.1098/rspb.2016.2758 PMC5394659 28404773

[38] McCormick, M. I., Fakan, E. P. & Palacios, M. M. Habitat degradation and predators have independent trait-mediated effects on prey. *Sci. Rep.* **9**, 15705 (2019). 31673067 10.1038/s41598-019-51798-2 PMC6823502

[39] McCormick, M. I. & Allan, B. J. Interspecific differences in how habitat degradation affects escape response. *Sci. Rep.* **7**, 426 (2017). 28348362 10.1038/s41598-017-00521-0 PMC5428724

[40] Ramasamy, R. A., Allan, B. J. & McCormick, M. I. Plasticity of escape responses: prior predator experience enhances escape performance in a coral reef fish. *PLoS One*. **10**, e0132790 (2015). 26244861 10.1371/journal.pone.0132790 PMC4526227

[41] Bostrom-Einarsson, L., Bonin, M. C., Munday, P. L. & Jones, G. P. Loss of live coral compromises predator-avoidance behaviour in coral reef damselfish. *Sci. Rep.* **8**, 7795 (2018). 29773843 10.1038/s41598-018-26090-4 PMC5958076

[42] Culp, J. M. et al. Incorporating traits in aquatic biomonitoring to enhance causal diagnosis and prediction. *Integr. Environ. Assess. Manage.* **7**, 187–197 (2011). 10.1002/ieam.128 21442732

[43] Pilière, A. et al. On the importance of trait interrelationships for understanding environmental responses of stream macroinvertebrates. *Freshwat Biol.* **61**, 181–194 (2016).

[44] De’Ath, G. Boosted trees for ecological modeling and prediction. *Ecology* **88**, 243–251 (2007). 17489472 10.1890/0012-9658(2007)88[243:btfema]2.0.co;2

[45] Elith, J., Leathwick, J. R. & Hastie, T. A working guide to boosted regression trees. *J. Anim. Ecol.* **77**, 802–813 (2008). 18397250 10.1111/j.1365-2656.2008.01390.x

[46] Buston, P. M. & Elith, J. Determinants of reproductive success in dominant pairs of clownfish: a boosted regression tree analysis. *J. Anim. Ecol.* **80**, 528–538 (2011). 21284624 10.1111/j.1365-2656.2011.01803.x

[47] Domenici, P. Context-dependent variability in the components of fish escape response: integrating locomotor performance and behavior. *J. Experimental Zool. Part. A: Ecol. Genet. Physiol.* **313**, 59–79 (2010). 10.1002/jez.580 20073047

[48] Kerrigan, B. Temporal patterns in size and condition at settlement in two tropical reef fishes (Pomacentridae: *Pomacentrus amboinensis* and *P. nagasakiensis*). *Mar. Ecol. Prog Ser.* **135**, 27–41 (1996).

[49] McCormick, M. I. & Molony, B. W. Quality of the reef fish Upeneus tragula (Mullidae) at settlement: is size a good indicator of condition? *Mar. Ecol. Prog. Ser.* **98**, 45–54 (1993).

[50] Ferrari, M. C., Wisenden, B. D. & Chivers, D. P. Chemical ecology of predator–prey interactions in aquatic ecosystems: a review and prospectus. *Can. J. Zool.* **88**, 698–724 (2010).

[51] Mitchell, M. D., Cowman, P. F. & McCormick, M. I. Are chemical alarm cues conserved within the coral reef fish family Pomacentridae? *PLoS One*. **7**, e47428 (2012). 23094047 10.1371/journal.pone.0047428 PMC3475700

[52] Lönnstedt, O. M., McCormick, M. I., Meekan, M. G., Ferrari, M. C. & Chivers, D. P. Learn and live: predator experience and feeding history determines prey behaviour and survival. *Proc. R. Soc. B* **279**, 2091–2098 (2012). 22237904 10.1098/rspb.2011.2516 PMC3321713

[53] McCormick, M. I., Ferrari, M. C. O., Fakan, E. P., Barry, R. P. & Chivers, D. P. Diet cues and their utility for risk assessment in degraded habitats. *Anim. Behav.* **152**, 19–28 (2019).

[54] Lönnstedt, O. M., McCormick, M. I., Chivers, D. P. & Ferrari, M. C. Habitat degradation is threatening reef replenishment by making fish fearless. *J. Anim. Ecol.* **83**, 1178–1185 (2014). 24498854 10.1111/1365-2656.12209

[55] McCormick, M. I., Chivers, D. P., Allan, B. J. M. & Ferrari, M. C. O. Habitat degradation disrupts neophobia in juvenile coral reef fish. *Global Change Biol.* **23**, 719–727 (2017). 10.1111/gcb.13393 27393344

[56] Lönnstedt, O. M., McCormick, M. I., Chivers, D. P. & Ferrari, M. C. O. Habitat degradation is threatening reef replenishment by making fish fearless. *J. Anim. Ecol.* **83**, 1178–1185 (2014). 24498854 10.1111/1365-2656.12209

[57] McCormick, M. I., Barry, R. & Allan, B. J. M. Algae associated with coral degradation affects risk assessment in coral reef fishes. *Sci. Rep.* **7**, 16937 (2017). 29208978 10.1038/s41598-017-17197-1 PMC5717098

[58] Coker, D., Wilson, S. & Pratchett, M. Importance of live coral habitat for reef fishes. *Rev. Fish. Biol. Fish.* **24**, 89–126 (2014).

[59] Meekan, M., Wilson, S., Halford, A. & Retzel, A. A comparison of catches of fishes and invertebrates by two light trap designs, in tropical NW Australia. *Mar. Biol.* **139**, 373–381 (2001).

[60] Rummer, J. L. et al. Life on the edge: thermal Optima for aerobic scope of Equatorial reef fishes are close to current day temperatures. *Global Change Biol.* **20**, 1055–1066 (2014). 10.1111/gcb.12455 PMC4677772 24281840

[61] McCormick, M. I. Behaviourally mediated phenotypic selection in a disturbed coral reef environment. *PLoS ONE*. **4**, e7096 (2009). 19763262 10.1371/journal.pone.0007096 PMC2740825

[62] McCormick, M. I. & Hoey, A. S. Larval growth history determines juvenile growth and survival in a tropical marine fish. *Oikos* **106**, 225–242 (2004).

[63] McCormick, M. I. & Meekan, M. G. The importance of attitude: the influence of behaviour on survival at an ontogenetic boundary. *Mar. Ecol. Prog Ser.* **407**, 173–185 (2010).

[64] Poulos, D. E. & McCormick, M. I. Asymmetries in body condition and order of arrival determine competitive ability and survival in a coral reef fish. *Oecologia* **179**, 719–728 (2015). 26220881 10.1007/s00442-015-3401-8

[65] Munday, P. L. et al. Replenishment of fish populations is threatened by ocean acidification. *PNAS* **107**, 12930–12934 (2010). 20615968 10.1073/pnas.1004519107 PMC2919925

[66] McCormick, M. I. et al. Microplastic exposure interacts with habitat degradation to affect behaviour and survival of juvenile fish in the field. *Proc. B*. **287**, 20201947 (2020). 10.1098/rspb.2020.1947 PMC7661286 33109008

[67] De’ath, G. & Fabricius, K. E. Classification and regression trees: a powerful yet simple technique for ecological data analysis. *Ecology* **81**, 3178–3192 (2000).

[68] Schapire, R. E. et al. *Nonlinear Estimation and Classification*, Vol. 171. *Lecture Notes in Statistics* (ed. Denison, D. D.) 149–171 (Springer, 2003).

[69] Doherty, P. J. et al. High mortality during settlement is a population bottleneck for a tropical surgeonfish. *Ecology* **85**, 2422–2428 (2004).

[70] Almany, G. R. & Webster, M. S. The predation gauntlet: early post-settlement mortality in coral reef fishes. *Coral Reefs*. **25**, 19–22 (2006).

[71] McCormick, M. I. & Holmes, T. H. Prey experience of predation influences mortality rates at settlement in a coral reef fish, *Pomacentrus amboinensis*. *J. Fish. Biol.* **68**, 969–974 (2006).

[72] Ferrari, M. C. O. et al. Living in a risky world: the onset and ontogeny of an integrated antipredator phenotype in a coral reef fish. *Sci. Rep.* **5**, 15537 (2015). 26515787 10.1038/srep15537 PMC4626771

[73] Rogers, L., Zucca, J. & Vallortigara, G. Advantages of having a lateralized brain. *Proc. R. Soc. B* **271**, S420–S422 (2004). 15801592 10.1098/rsbl.2004.0200 PMC1810119

[74] Rogers, L. J. Brain lateralization and cognitive capacity. *Animals* **11**, 1996 (2021). 34359124 10.3390/ani11071996 PMC8300231

[75] Chivers, D. P. et al. At odds with the group: changes in lateralization and escape performance reveal conformity and conflict in fish schools. *Proc. R. Soc. B.* **283**, 20161127 (2016). 10.1098/rspb.2016.1127 PMC5095373 27798294

[76] Dadda, M., Koolhaas, W. H. & Domenici, P. Behavioural asymmetry affects escape performance in a teleost fish. *Biol. Lett.* **6**, 414–417 (2010). 20089537 10.1098/rsbl.2009.0904 PMC2880054

[77] Ferrari, M. C. et al. The effects of background risk on behavioural lateralization in a coral reef fish. *Funct. Ecol.* **29**, 1553–1559 (2015).

[78] Ferrari, M. C. et al. Daily variation in behavioural lateralization is linked to predation stress in a coral reef fish. *Anim. Behav.* **133**, 189–193 (2017).

[79] Fuiman, L. A., Meekan, M. G. & McCormick, M. I. Maladaptive behavior reinforces a recruitment bottleneck in newly settled fishes. *Oecologia* **164**, 99–108 (2010). 20602117 10.1007/s00442-010-1712-3

[80] Fuiman, L. A., Rose, K. A., Cowan Jr, J. H. & Smith, E. P. Survival skills required for predator evasion by fish larvae and their relation to laboratory measures of performance. *Anim. Behav.* **71**, 1389–1399 (2006).

[81] McCormick, M. I., Fakan, E. P. & Palacios, M. M. Habitat degradation and predators have independent trait-mediated effects on prey. *Sci. Rep.* **9**, 15705 (2019). 31673067 10.1038/s41598-019-51798-2 PMC6823502

[82] Domenici, P. & Blake, R. The kinematics and performance of fish fast-start swimming. *J. Exp. Biol.* **200**, 1165–1178 (1997). 9319004 10.1242/jeb.200.8.1165

[83] McCormick, M. I. & Allan, B. Interspecific differences in how habitat degradation affects escape response. *Sci. Rep.* **7**, 426 (2017). 28348362 10.1038/s41598-017-00521-0 PMC5428724

[84] Fakan, E. P., Allan, B. J. M., Illing, B., Hoey, A. S. & McCormick, M. I. Habitat complexity and predator odours impact on the stress response and antipredation behaviour in coral reef fish. *PLoS One* **18**, e0286570 (2023). 37379294 10.1371/journal.pone.0286570 PMC10306203

[85] Ydenberg, R. C. & Dill, L. M. The economics of fleeing from predators. *Adv. Study Behav.* **16**, 229–249 (1986).

[86] Holmes, T. H. & McCormick, M. I. Location influences size-selective predation on newly settled reef fish. *Mar. Ecol. Prog Ser.* **317**, 203–209 (2006).

[87] Holmes, T. & McCormick, M. I. Size-selectivity of predatory reef fish on juvenile prey. *Mar. Ecol. Prog Ser.* **399**, 273–283 (2010).

[88] Meekan, M. G., von Kuerthy, C., McCormick, M. I. & Radford, B. Behavioural mediation of the costs and benefits of fast growth in a marine fish. *Anim. Behav.* **79**, 803–809 (2010).

[89] McCormick, M. I. & Weaver, C. J. It pays to be pushy: intracohort interference competition between two reef fishes. *PloS One*. **7**, e42590 (2012). 22900030 10.1371/journal.pone.0042590 PMC3416846

[90] Palacios, M. D. & McCormick, M. I. Positive indirect effects of top-predators on the behaviour and survival of juvenile fishes. *Oikos* **130**, 219–230 (2021).

[91] Ahti, P. A., Kuparinen, A. & Uusi-Heikkilä, S. Size does matter — the eco-evolutionary effects of changing body size in fish. *Environ. Rev.* **28**, 311–324 (2020).

[92] Sogard, S. M. Size-selective mortality in the juvenile stage of teleost fishes: A review. *Bull. Mar. Sci.* **60**, 1129–1157 (1997).

[93] McCormick, M. I. & Meekan, M. G. Social facilitation of selective mortality. *Ecology* **88**, 1562–1570 (2007). 17601147 10.1890/06-0830

[94] Richardson, L. E. et al. Examining current best-practices for the use of wild post-larvae capture, culture, and release for fisheries enhancement. *Front. Mar. Sci.* **9**, 1058497 (2023).

[95] Mirza, R. S. & Chivers, D. P. Predator-recognition training enhances survival of brook trout: evidence from laboratory and field-enclosure studies. *Can. J. Zool.* **78**, 2198–2208 (2000).

[96] Mitchell, M. D., McCormick, M. I., Ferrari, M. C. O. & Chivers, D. P. Generalization of learned predator recognition in coral reef ecosystems: how cautious are damselfish? *Funct. Ecol.* **27**, 299–304 (2013).

[97] Ferrari, M. C., Gonzalo, A., Messier, F. & Chivers, D. P. Generalization of learned predator recognition: an experimental test and framework for future studies. *Proc. R. Soc. B* **274**, 1853–1859 (2007). 17519190 10.1098/rspb.2007.0297 PMC2270927

[98] Lönnstedt, O. M. & McCormick, M. I. Damsel in distress: captured damselfish prey emit chemical cues that attract secondary predators and improve escape chances. *Proc. R. Soc. B* **282**, 20152038 (2015). 26511043 10.1098/rspb.2015.2038 PMC4650161

[99] Crane, A. L. & Ferrari, M. C. O. *Social Learning Theory: Phylogenetic Considerations across animal, plant, and Microbial Taxa *(eds Clark, K. B.) 53–82 (Nova Science, 2013).

