# Supplementary material for: Habitat degradation alters trait-based survival in a coral reef fish
Source: Sci Rep. 2025 Nov 4;15:38631. doi: 10.1038/s41598-025-22578-y (PMC12586590; doi:10.1038/s41598-025-22578-y)
Supplement: Supplementary file 1 — Supplementary Material 1 [file 41598_2025_22578_MOESM1_ESM.pdf]

SUPPLEMENTARY FILE

Experimental protocols

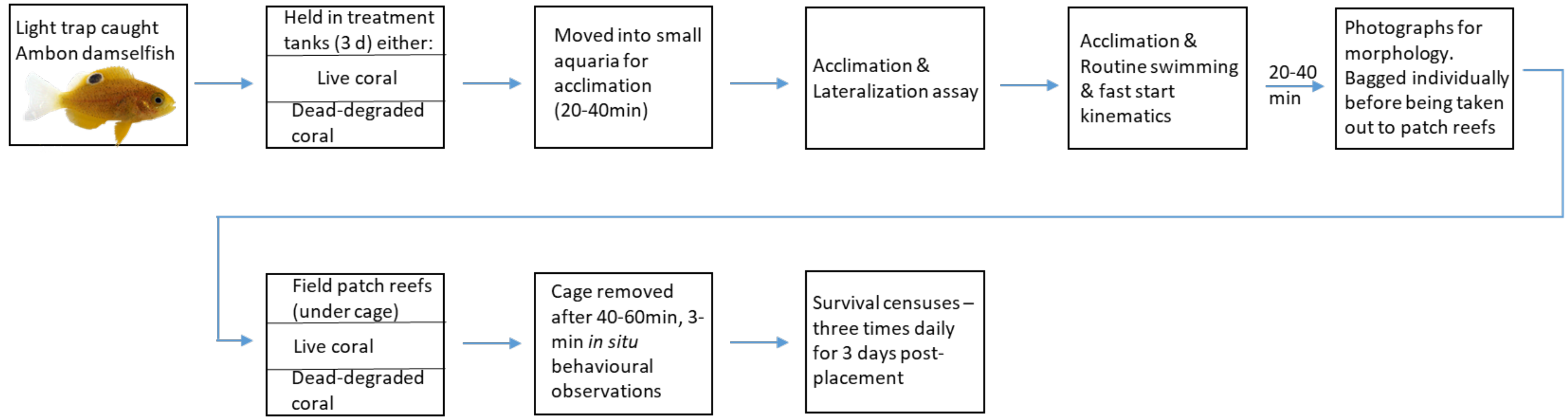

Figure S1. Schematic diagram of the order and timing of experimental procedures conducted in this study. Juvenile Ambon damselfish (*Pomacentrus amboinensis*) were caught using light traps and reared for three days in treatment tanks fed from saltwater sources of either water that had passed over live or dead-degraded coral (*Pocillopora damicornis*). On trial days, were put through performance assays to assess lateralization, routine swimming and fast-start kinematics, and then photographed and bagged, before being released onto coral patch reefs (live or dead-degraded *Poc. damicornis*) where behaviour and survival were monitored (Photographic credit M. McCormick).

Study species and site

Pomacentrus amboinensis is a site-attached habitat generalist with juveniles and adults frequently associating with rubble, live and dead coral patches (McCormick 2016), although it preferentially settles on live coral (McCormick and Weaver 2012). Settlement follows a 15-23 day larval phase (Kerrigan 1996), with newly settled individuals (~10-12mm SL) rapidly exposed to a range of predators including lizardfish (*Synodus dermatogenys*), small cod (*Cephalopholis microprion*), dottybacks (*Pseudochromis fuscus*) and moon wrasse (*Thalassoma lunare*) that will quickly prey on individuals that venture too far from shelter (Holmes and McCormick 2010).

Laboratory measures of morphology and performance

The experimental protocols followed in this study were similar to and based on those performed by McCormick et al. (2018). The sequence of assessments performed on trial days was as follows (Fig. S1). On the morning of assessment, fish were carefully transferred individually using a fine mesh net from the treatment tanks into small individual labelled aquaria held in a flow-through water bath to maintain water temperature (26-28°C) to habituate to isolation. Twenty to forty minutes later, the fish was carefully transferred to the lateralization chamber where they were left for a further three minutes to habituate to the apparatus, before being tested in a detour test (n = 10 turns; McCormick et al. (2018)). After being assessed for laterality, they were returned to their individual aquaria (in water bath) and left for ten minutes before being carefully transferred to the fast-start arena and left for a further five minutes to habituate. The fish were then filmed at 30fps for two minutes for a routine swimming assessment, after which a repeatable drop stimulus was used to elicit a fast-start response (filmed at 480fps; (Allan et al. 2020)). After routine swimming and fast-start assessment, the fish were returned to their individual aquaria (in water bath) for twenty to forty minutes before being placed into labelled small clip-seal plastic bag of aerated seawater and photographed laterally against 0.5cm grid. These photographs were then analysed to measure the fish's morphological features ((1) body size (standard length and body depth), (2) lateral body area, (3) relative size of the ocellus (false eye spot)). The details of each assay are given below.

Morphology

Lateral photographs were taken of each individual fish against a 50mm grid background and morphology was quantified from these photos using the image-analysis package Image-J (imagej.nih.gov/ij/). The morphological variables measured were standard length, body depth, lateral body area, whole ocellus area, black ocellus area, eye area and pupil area (Fig. S2). From the measurements of the whole ocellus and eye area, the relative size of the ocellus to eye (ocellus/eye area) was calculated. Total fin area, while a potentially useful variable given its use in predicting swimming speed/performance (e.g. Fisher and Hogan (2007)), was not measured as the slightly-transparent caudal fin was difficult to discern from the white, gridded background of the photographs.

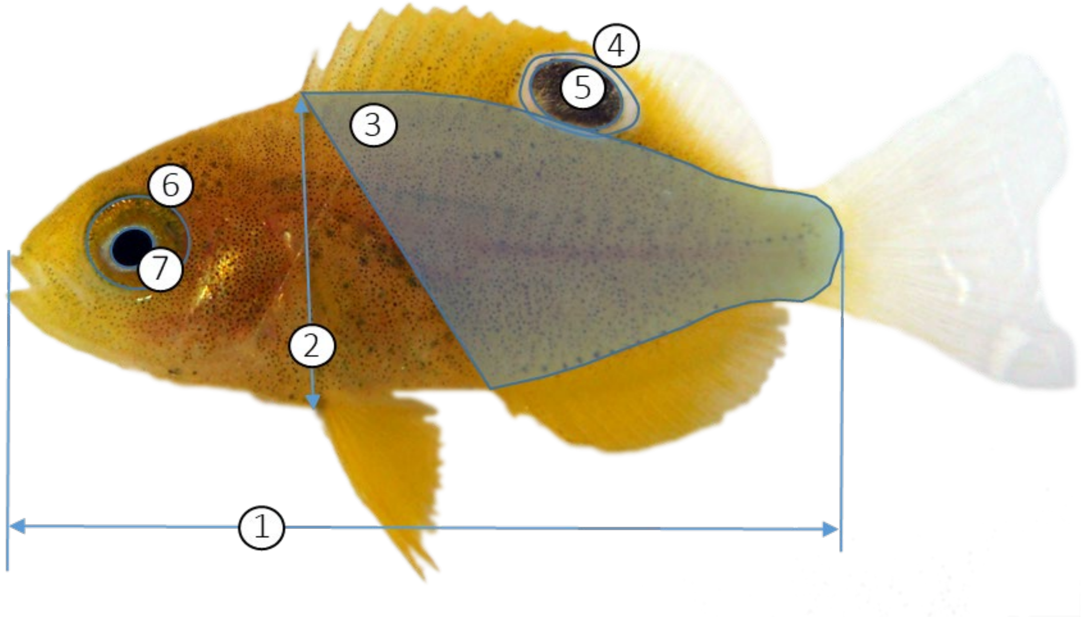

Figure S2. Labelled photograph of juvenile Ambon damselfish (*P. amboinensis*) showing measured morphological traits: (1) standard length, (2) body depth, (3) lateral body area, (4) whole ocellus area, (5) black ocellus area, (6) eye area, and (7) pupil area (photo credit: M. McCormick).

Lateralization

Lateralization, the specialization of the left and right hemispheres of the brain for different functions, provides a wide range of ecological benefits to numerous organisms, from fish to

primates (Vallortigara and Rogers 2005). This asymmetry allows for more efficient processing of information and the ability to perform multiple tasks simultaneously, which can be crucial for survival and reproduction. Some of the key ecological benefits include: predator vigilance and foraging, group coordination and social behaviour, prey capture and hunting, navigation and spatial orientation, and escape responses.

Laterality of each fish was determined using a detour test (Bisazza et al. 1998). The apparatus (lateralization chamber) consisted of a grey-coloured PVC tank (600mm length x 300mm width x 154mm height), with a runway in the middle (250mm length x 30 mm width x 120mm height) and grey (same colour) PVC barriers positioned perpendicular to the orientation of the runway at both ends (30mm ahead of the runway) (Fig. S3). Water (same seawater source as treatment) in the chamber was 60mm deep and was changed every four trials to avoid changes in water temperature (26-28°C) and dissolved oxygen levels in the arena.

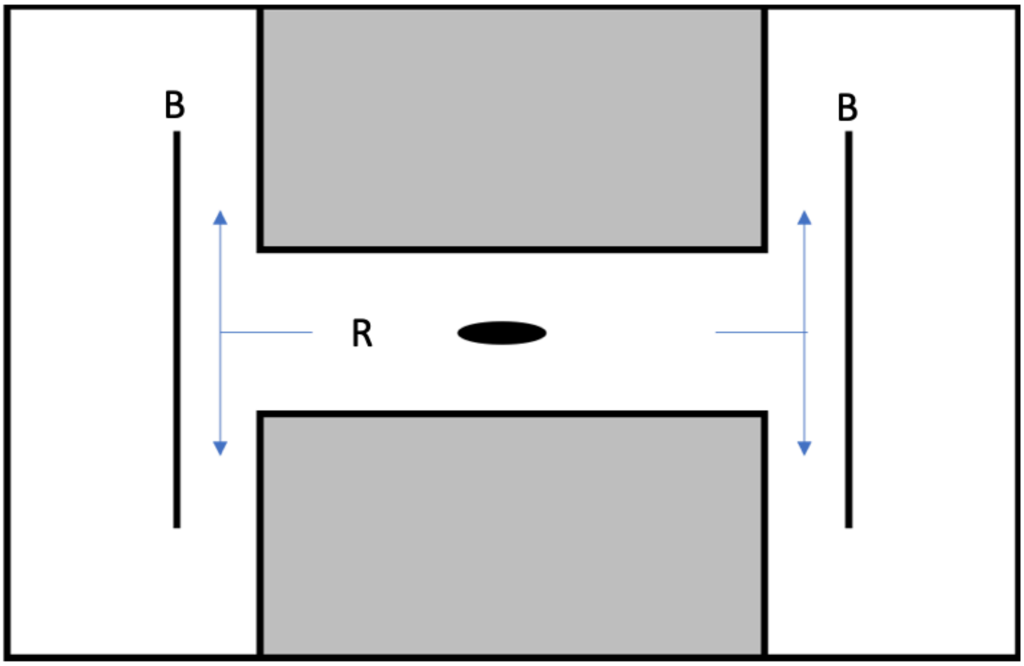

Figure S3. Schematic of lateralization chamber (R = runway, B = barrier, black oval indicates fish; schematic modified from Bisazza et al. (1998)).

At the start of each trial, individual fish were introduced into the middle of the runway and left for two minutes to habituate. During the trial, the fish were gently manoeuvred to the starting point of the runway. The fish then swam along the runway until it faced the barrier,

where it then made a decision to turn left or right around the barrier. This was repeated ten times (ten consecutive replicates per fish) from alternate ends of the runway in order to account for any potential asymmetry in the setup, and in order to avoid the fish taking a ‘familiar’ route the fish entered the runway from the opposite side to which they exited. Turning was recorded by direct observation, and the criterion used was the first direction taken by the fish when exiting the runway.

Two different calculations of lateralization were computed. Relative laterality was used to quantify the level of turning bias according to the following formula (Bisazza et al. 1998):

$$\text{Relative lateralization} = \frac{\text{number of right turns} - \text{number of left turns}}{\text{number right turns} + \text{number of left turns}} \times 100$$

This yields a value between -100 (highly lateralised to the left) and 100 (highly lateralised to the right).

Absolute laterality was also calculated to determine the strength of an individual’s lateralization regardless of their preference for left or right (Ferrari et al. 2015; Chivers et al. 2016):

$$\text{Absolute lateralization} = \left| \frac{\text{number of right turns} - \text{number of left turns}}{\text{total number of trials}} \times 100 \right|$$

This yields a value between zero (no side preference; not lateralised) and 100 (absolute side preference, regardless of left/right; highly lateralised).

Routine swimming and fast-start protocol

Routine swimming and fast-start kinematics were examined using the following set up: a transparent circular acrylic arena (200mm diameter; 80mm height) within a large opaque-sided plastic tank (585 x 420 x 330 mm) with a transparent Perspex bottom on top of a box open at one side with a mirror at a 45° angle to allow behaviour and responses to be filmed from below using the fishes silhouette (see Fig. S2 in McCormick et al. 2018). The water level was maintained at 60mm to reduce movements in the vertical plane. For each replicate the water in the arena was replaced with fresh seawater (of same type as fish’s treatment) to

maintain water quality and temperature (26-28°C). The arena was illuminated by a LED light strip wrapped around the outside of the opaque holding tank. The camera used for these assays was a Casio Exilim ZR (EX-ZR1000).

At the beginning of the trial, individual fish were placed inside the acrylic testing arena and allowed to acclimate for five minutes. After the five-minute habituation period routine swimming was recorded as a silhouette from below at 30fps for two minutes. A fast-start was then stimulated by the release of a conical weight with a tapered end into the testing arena and recorded at 480fps. The weight was dropped into the arena via release from an electromagnet and attached to a piece of fishing line that was long enough to ensure that the tapered tip of the weight only just touched the surface of the water. Fish were only startled when they had moved into the middle portion of the arena, as this allowed the individual to move equal distance in any direction and standardizing for fish position relative to the position of the stimulus (weight). To avoid a premature fast-start response associated with visual stimulation occurring, the stimulus was released from above into a piece of 48.5mm diameter PVC pipe with the bottom edge 10mm above the water level. To ensure standardised protocols, fast-start variables were only measured when the fish performed a C-start (body takes on shape of a 'C') indicating the start of an escape response (Domenici and Blake 1997).

The routine swimming and fast-start variables were analysed using Image-J with a manual tracking plugin, and the centre of mass (CoM) of the fish was tracked for the duration of the responses. Routine swimming was analysed from the two minute, 30fps video sequences and measured by tracking the position of fish's CoM every second, resulting in 120 data points. From these data points, the distance (meters) covered by the fish, average speed (ms^{-1}) and maximum speed (ms^{-1}) could be calculated. For the fast-start videos, the original 480fps videos were trimmed and exported as an image sequence at 30fps. Four kinematic variables were measured for the fast-start response: (1) response latency (s) – the time interval between the onset of the stimulus and the first detectable movement leading to the escape of the animal (beginning of the C-start); (2) response distance (m) – the total distance covered by the fish during the initial escape response (first 20 frames post beginning of C-start); (3) response speed ($m.s^{-1}$) – average speed reached during the initial escape response (first 20 frames post beginning of C-start); and (4) maximum response speed ($m.s^{-1}$) – the maximum speed achieved at anytime during the initial escape behaviour (first 20 frames post beginning of C-start). To standardise for variation in the position of the fish relative to the stimulus, the distance between the stimulus and the fish's CoM was also measured.

Behavioural measures and survival in the field

Individual fish were placed onto uniquely numbered habitat patches (25 x 20 x 20 cm) constructed of either healthy, live or dead-degraded *Poc. damicornis* depending on the seawater source they had been conditioned with in the laboratory (i.e., individuals from the live coral treatment were placed onto live coral patches, and those from the dead-degraded coral treatment were placed on dead-degraded coral patches). The patches were located on the sand approximately 3m from the edge of a shallow reef (Sunbird Reef, Lizard Island) and approximately 4m apart to minimise fishes moving between patches. Live and dead coral patches were alternated along the reef edge. Prior to the introduction of the focal fish, all resident fishes and mobile invertebrates were removed from the patch reefs using a hand net. After placing the fish on the patch, a small cage (30 x 30 x 30 cm made with 12mm square mesh) was placed over the patch for 40-60 minutes to allow the fish to habituate to the patches without the threat of predation. Previous studies have shown that individually tagged settlement-stage damselfish do not move between patches and that losses from patch reefs is due to predation (Hoey and McCormick 2004; McCormick and Meekan 2007; McCormick 2009), therefore movement is not likely to be a significant source of bias and fishes in this study were not tagged in order to minimize stress associated with handling. Recruitment of *P. amboinensis* was relatively low to the fringing reef used for the field component of the study and there was no instance where more than one *P. amboinensis* was found on of the monitored patch reefs during the study period.

The behaviour of each fish was assessed by a SCUBA diver (MIM) positioned ~1.5 m away from the patch. Three aspects of activity and behaviour were assessed: a) bite rate; b) total distance moved (cm); c) maximum distance ventured from the habitat patch (cm). Total distance moved and maximum distance ventured were estimated by keeping track of where the fish travelled and from knowing the measured dimensions of the patch reef. Our previous research has demonstrated that individually tagged recruit-sized damselfishes do not generally move between patch reefs, and any loss from these patches is primarily due to predation (McCormick and Meekan 2007; McCormick 2009). For example, in one study, 20 elastomer-tagged, newly settled damselfish were released across 15 patch reefs spaced 8 meters apart. The color differences in the tags between adjacent reefs allowed for tracking movement, and the study found that only 3 out of 300 fish (1%) moved to neighboring reefs over a 3-day period (Hoey & McCormick 2004). This suggests that movement does not significantly impact the results of the current study. In our study, fish generally began feeding

within 30 seconds of release, indicating that despite the stress from prior performance assessments, they quickly acclimated to their new environment and experienced minimal stress.

Table S1. Descriptive statistics of raw data for each of the morphological, performance (kinematic, routine swimming, lateralization) and behavioural variables measured in this study. The variables included in final analyses are indicated by an asterisk after their name. Variables with superscript 1 were standardised by distance from the startle stimulus. Examples are given of previous studies that link these variables to survival.

Variable Type	Variable	Mean	Std Dev	Min, Max	CV	Transformation	Examples of studies linking variables to survival
Morphological	Standard length (mm)*	12.59	0.514	11.40, 14.37	4.082	-	Holmes and McCormick (2009); Holmes and McCormick (2010); Perez and Munch (2010); Fakan et al. (2024)
	Body depth (mm)*	5.023	0.265	4.412, 5.894	5.276	-	
	Ocellus/eye area*	0.871	0.166	0.524, 1.393	19.059	$Log_{10}(x)$	Lönnstedt et al. (2013); Fakan et al. (2024)
	Lateral body area (mm^2)*	22.62	2.104	18.10, 29.12	9.302	-	Kern et al. (2016)
Kinematic (Fast-start)	Response latency (s)*[1]	0.0270	0.0266	0.0083, 0.252	98.519	$1/\sqrt{x}$)	Katzir and Camhi (1993); Fuiman et al. (2006); Domenici (2010); Allan et al. (2015); McCormick et al. (2018)
	Response average speed ($m.s^{-1}$)*[1]	0.722	0.171	0.262, 1.275	23.684	-	
	Response maximum speed ($m.s^{-1}$)*[1]	1.361	0.349	0.547, 3.396	25.643	$\sqrt{x}$	
	Response distance (m)	0.018	0.0042	0.0065, 0.0306	23.333	-	
Routine swimming	Average Speed ($m.s^{-1}$)*	0.0037	0.0031	0.00019, 0.0294	87.784	$Log_{10}(x)$	Fuiman et al. (2006); Fuiman et al. (2010)
	Maximum Speed ($m.s^{-1}$)*	0.0193	0.0119	0.0012, 0.0718	62.658	$Log_{10}(x)$	
	Distance (m)[1]	0.446	0.366	0.0224, 3.531	82.063	-	
Lateralization	Absolute lateralization	31.33	25.77	0, 100	82.253	-	Ferrari et al. (2015); Chivers et al. (2016)
	Relative lateralization*	16.11	37.27	-100, 100	231.347	-	Bisazza et al. (1998)
Behavioural	Bite rate (bites/3min)*	32.92	21.58	0, 90	65.553	$x^{0.7}$	McCormick (2009); McCormick et al. (2018)
	Total distance moved (cm)*	19.11	12.22	2, 60	63.946	$\sqrt{x}$	Lönnstedt et al. (2012)
	Maximum distance ventured (Max DV, cm)*	4.212	3.781	0, 16	89.767	-	McCormick (2012)

Table S2. Correlation matrix showing statistically significant ($p < 0.05$) correlations between transformed variables.

Variable type	Morphology				Kinematic			Routine		Lateralization	Field behaviour			
Variable	SL	Body depth	Ocellus/ pupil	Lateral body area	Latency	Response speed	Max response speed	Routine speed	Routine max speed	Relative lateralization	Bite rate	Total distance moved	Max distance ventured	PC1 behaviour
SL		0.77		0.78		0.3	0.31							
Body depth	0.77			0.76	0.13	0.27	0.21							
Ocellus/pupil				0.28		0.15								
Lateral body area	0.78	0.76	0.28			0.25	0.23							
Latency		0.13				0.4	0.24							
Response speed	0.3	0.27	0.15	0.25	0.4		0.69							
Max response speed	0.31	0.21		0.23	0.24	0.69		0.17						
Routine speed							0.17		0.86			0.16		0.15
Routine max speed								0.86						
Relative lateralization														
Bite rate												0.55	0.39	0.78
Total distance moved								0.16			0.55		0.59	0.88
Max distance ventured											0.39	0.59		0.8
PC1 behaviour								0.15			0.78	0.88	0.8	

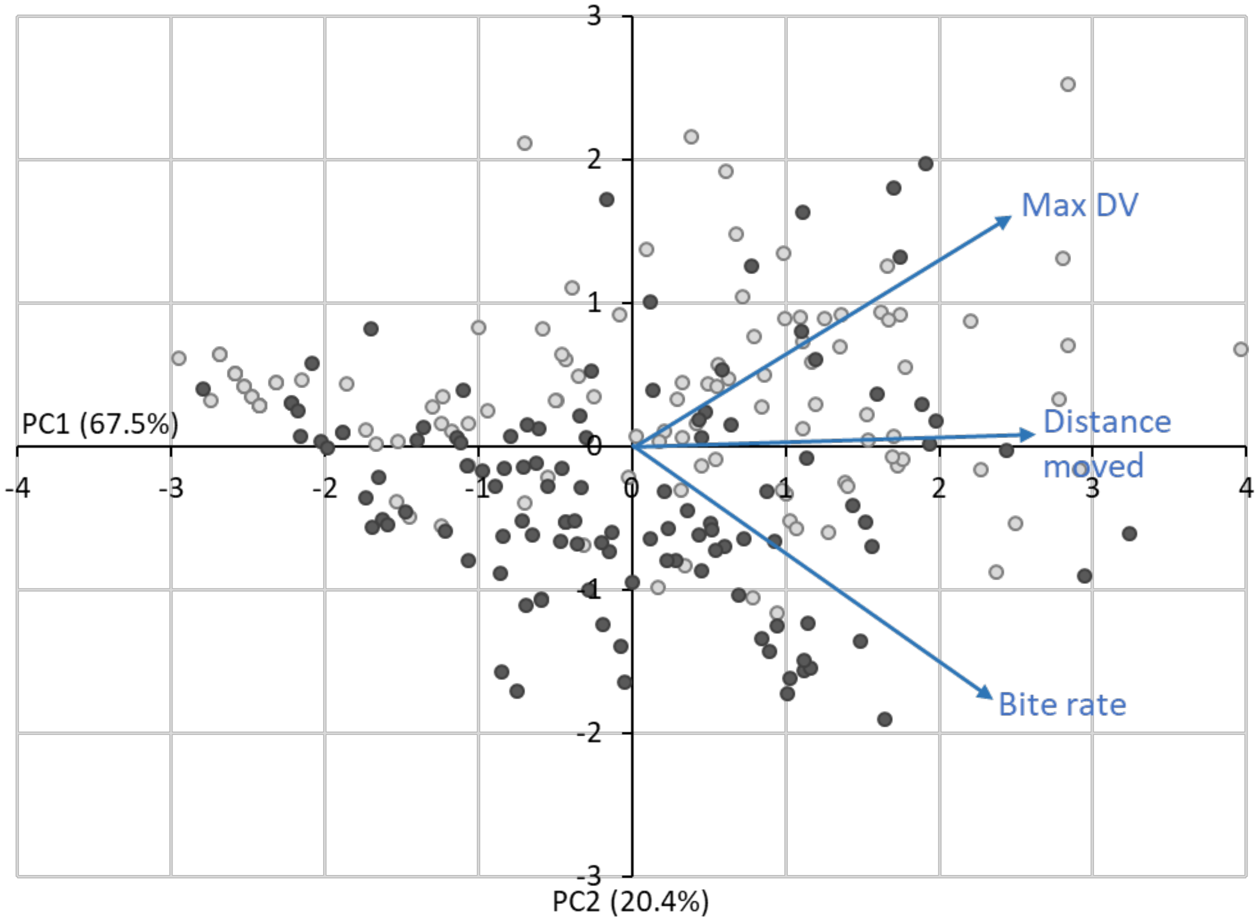

Figure S4. Biplot of a principal component analysis (PCA) showing the distribution of individual fish relative to bite rate, total distance moved, and maximum distance ventured from 3 min behavioural assessments. The biplot illustrates the relationships between the individual fish and the original behavioural variables. Each point represents an individual fish, and the arrows indicate the direction and strength of the original variables in the new principal component space. The length of an arrow is proportional to the variable's contribution to the PCA, and the angle between the arrows reflects the correlation between the variables. Variables with a small angle between them (e.g., distance moved and maximum distance ventured) are highly correlated. The positioning of a point relative to the arrows indicates the fish's score on that variable. For example, fish located in the direction of the "Bite rate" arrow exhibited higher bite rates. The first two principal components (PC1 and PC2) are displayed, which together account for a significant portion of the total variance in the dataset (87.9%). Fish from dead-degraded coral is light grey, while fish from live coral are represented as black dots.

Supplementary file references

Allan BJ, Domenici P, Munday PL, McCormick MI (2015) Feeling the heat: the effect of acute temperature changes on predator–prey interactions in coral reef fish. Conservation Physiology 3:cov011

Allan BJM, Illing B, Fakan E, Narvaez P, Grutter AS, Sikkel PC, McClure E, Rummer J, McCormick MI (2020) Parasite infection directly impacts escape response and stress levels in fish. J Exp Biol jeb.230904

Bisazza A, Facchin L, Pignatti R, Vallortigara G (1998) Lateralization of detour behaviour in poeciliid fish: the effect of species, gender and sexual motivation. Behavioural Brain Research 91:157-164

Chivers DP, McCormick MI, Allan BJ, Mitchell MD, Gonçalves EJ, Bryshun R, Ferrari MC (2016) At odds with the group: changes in lateralization and escape performance reveal conformity and conflict in fish schools. Proceedings of the Royal Society B: Biological Sciences 283:20161127

Domenici P (2010) Context-dependent variability in the components of fish escape response: integrating locomotor performance and behavior. Journal of Experimental Zoology Part A: Ecological Genetics and Physiology 313:59-79

Domenici P, Blake R (1997) The kinematics and performance of fish fast-start swimming. J Exp Biol 200:1165-1178

Fakan EP, McCormick MI, Jones GP, Hoey AS (2024) Habitat and morphological characteristics affect juvenile mortality in five coral reef damselfishes. Coral Reefs 43:171-183

Ferrari MC, McCormick MI, Allan BJ, Choi RB, Ramasamy RA, Chivers DP (2015) The effects of background risk on behavioural lateralization in a coral reef fish. Funct Ecol 29:1553-1559

Fisher R, Hogan JD (2007) Morphological predictors of swimming speed: a case study of pre-settlement juvenile coral reef fishes. Journal of Experimental Biology 210:2436-2443

Fuiman LA, Meekan MG, McCormick MI (2010) Maladaptive behavior reinforces a recruitment bottleneck in newly settled fishes. Oecologia 164:99-108

Fuiman LA, Rose KA, Cowan Jr JH, Smith EP (2006) Survival skills required for predator evasion by fish larvae and their relation to laboratory measures of performance. Anim Behav 71:1389-1399

Hoey AS, McCormick MI (2004) Selective predation for low body condition at the larval-juvenile transition of a coral reef fish. Oecologia 139:23-29

Holmes T, McCormick MI (2010) Size-selectivity of predatory reef fish on juvenile prey. Mar Ecol Prog Ser 399:273-283

Holmes TH, McCormick MI (2009) Influence of prey body characteristics and performance on predator selection. Oecologia 159:401-413

Katzir G, Camhi JM (1993) Escape response of black mollies (*Poecilia sphenops*) to predatory dives of a pied kingfisher (*Ceryle rudis*). Copeia 1993:549-553

Kern EM, Robinson D, Gass E, Godwin J, Langerhans RB (2016) Correlated evolution of personality, morphology and performance. Anim Behav 117:79-86

Kerrigan B (1996) Temporal patterns in size and condition at settlement in two tropical reef fishes (Pomacentridae: *Pomacentrus amboinensis* and *P. nagasakiensis*). Mar Ecol Prog Ser 135:27-41

Lönnstedt OM, McCormick MI, Chivers DP (2013) Predator-induced changes in the growth of eyes and false eyespots. Scientific Reports 3:2259

Lönnstedt OM, McCormick MI, Meekan MG, Ferrari MC, Chivers DP (2012) Learn and live: predator experience and feeding history determines prey behaviour and survival. Proceedings of the Royal Society B: Biological Sciences 279:2091-2098

McCormick MI (2009) Behaviourally mediated phenotypic selection in a disturbed coral reef environment. PLoS ONE 4:e7096

McCormick MI (2012) Lethal effects of habitat degradation on fishes through changing competitive advantage. Proceedings of the Royal Society B: Biological Sciences 279:3899-3904

McCormick MI (2016) Protogyny in a tropical damselfish: females queue for future benefits. PeerJ 4:e2198

McCormick MI, Meekan MG (2007) Social facilitation of selective mortality. Ecology 88:1562–1570

McCormick MI, Weaver CJ (2012) It pays to be pushy: intracohort interference competition between two reef fishes. PloS One 7:e42590

McCormick MI, Fakan E, Allan BJM (2018) Behavioural measures determine survivorship within the hierarchy of whole-organism phenotypic traits. Funct Ecol 32:958–969

Perez KO, Munch SB (2010) Extreme selection on size in the early lives of fish. Evolution: International Journal of Organic Evolution 64:2450-2457

Vallortigara G, Rogers L (2005) Survival with an asymmetrical brain: advantages and disadvantages of cerebral lateralization. Behavioral and Brain Sciences 28:575-633
